# Ovarian cryopreservation after laparoscopic ovariectomy using the Endo-GIA stapling device and LAPRO-clip absorbable ligating clip in a woman: a case report

**DOI:** 10.1186/1752-1947-5-48

**Published:** 2011-02-03

**Authors:** Isabelle Roux, Michaël Grynberg, Jenna Linehan, Alexandra Messner, Xavier Deffieux

**Affiliations:** 1 AP-HP, Service de Gynécologie-Obstétrique et Médecine de la Reproduction, Hôpital Antoine Béclère, Clamart, F-92141, France; 2 Univ Paris-Sud, Faculté de Médecine Paris Sud, Le Kremlin Bicêtre, F-94275, France; 3 AP-HP, Department of Reproductive Biology, Antoine Béclère Hospital, Clamart, F-92141, France; 4 ER6, UPMC, Paris, F-75013, France

## Abstract

**Introduction:**

Several options are available for preserving fertility before cytotoxic treatment, including ovarian tissue cryopreservation. Most reported surgical techniques include electrocoagulation. Our hypothesis is that avoidance of electrocoagulation may decrease ovarian cortex injury during cryopreservation procedures.

**Case presentation:**

We report a laparoscopic technique of whole-ovary removal without coagulation using Endo-GIA forceps and clips. Laparoscopic ovariectomy was performed for cryopreservation in a 37-year-old Caucasian woman with breast cancer and for whom chemotherapy was planned. The procedure was completed quickly and without complication. This Endo-GIA procedure was of short duration with a short period of ischemia before freezing.

**Conclusion:**

Laparoscopic ovariectomy using the Endo-GIA stapling device procedure without coagulation may diminish ovary injury before ovarian cryopreservation.

## Introduction

Several options are available for preserving fertility before cytotoxic treatment, namely embryo cryopreservation, oocyte cryopreservation and ovarian tissue cryopreservation. Embryo cryopreservation results in good pregnancy rates, but the patient needs to be of pubertal or postpubertal age, have a partner and be able to undergo a cycle of ovarian stimulation [1]. Ovarian stimulation is not possible when chemotherapy cannot be delayed or when stimulation is contraindicated. Ovarian tissue transplantation after cryopreservation is an option despite being an experimental technique with few live births reported [2]. Either a part of cortical tissue [3] or whole ovary can be removed.

## Case presentation

**Figure 1:**
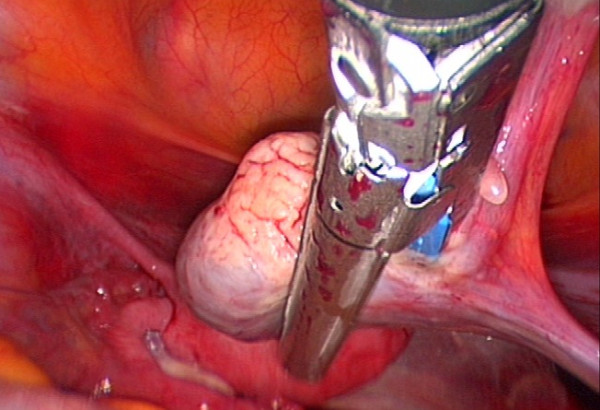
**The Endo-GIA Roticulator is used to cut the infundibulopelvic ligament and mesovarium**.

**Figure 2:**
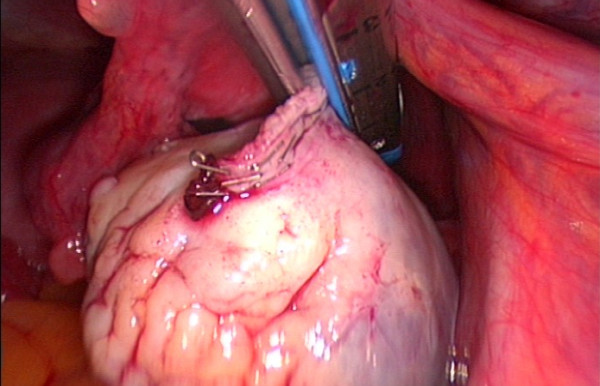
**Cutting the mesovarium using the Endo-GIA stapling device**.

**Figure 3:**
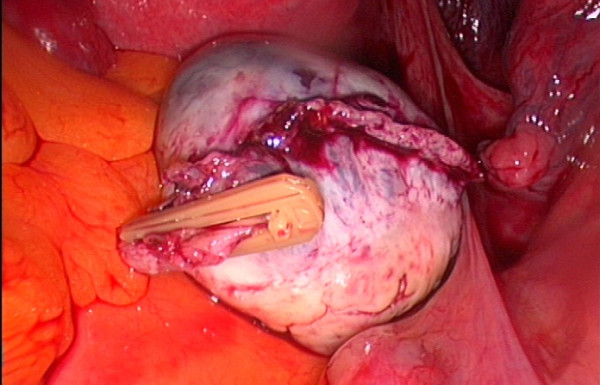
**Clamping the utero-ovarian ligament using vascular absorbable clips**.

We report a laparoscopic ovariectomy technique performed for cryopreservation in a 37 year-old Caucasian woman with breast cancer and for whom chemotherapy was planned. Despite being informed of the poor outcome in women of her age, this woman elected to undergo combined techniques for fertility preservation. As an adjuvant to the tissue preservation, immature oocyte retrieval was performed one day before the surgery. During laparoscopy, the ureter and the iliac vessels were identified. Through the medial 12-mm trocar, the Endo-GIA Roticulator (Tyco Healthcare, Covidien, Elancourt, France) was used to cut the infundibulopelvic ligament and mesovarium (Figures 1 and 2). The utero-ovarian ligament was then clamped with two vascular absorbable clips (Figure 3). The removed ovary was immediately handed over to a biologist team that was present in the operating room. No complications were reported from this procedure. Pathology revealed "normal" ovarian tissue. Our hypothesis is that avoidance of electrocoagulation may decrease ovarian cortex injury during cryopreservation procedure. There are no precise data demonstrating that electrocoagulation causes damage to the ovarian tissue in the course of ovarian tissue harvesting and cryopreservation. However, many experimental studies have shown that electrocoagulation (monopolar and bipolar energies) may be associated with damage to ovarian tissue. For example, ovarian drilling, especially bipolar electrocoagulation, causes extensive destruction of the ovary [4]. Furthermore, some data suggest that bipolar electrocoagulation of the ovarian parenchyma during laparoscopic ovarian cystectomy adversely affects ovarian function [5,6]. These data show possible impact of electrocoagulatory ovarian tissue damage on the outcome of ovarian tissue harvesting and reimplantation. Further studies should assess ovarian tissue damage and the results of ovarian cryopreservation according to the ovarian removal procedure (Endo-GIA vs. electrocoagulatory).

This Endo-GIA procedure was of short duration with a short period of ischemia before freezing. Furthermore, it is known that the Endo-GIA stapling device requires significantly less time than electrocoagulation [7]. However, some centers do not remove a whole ovary for ovarian tissue cryopreservation; instead, they remove only half to two-thirds of one macroscopically normal ovary's cortex. The Endo-GIA removal procedure cannot be used in these cases.

## Conclusion

Laparoscopic ovariectomy using the Endo-GIA stapling device procedure without coagulation is an optional ovariectomy technique that should diminish ovary injury before ovarian cryopreservation.

## Competing interests

The authors declare that they have no competing interests.

## Consent

Written informed consent was obtained from the patient for publication of this case report and accompanying images. A copy of the written consent is available for review from the Editor-in-Chief of this journal.

## Authors' contributions

Each author participated sufficiently in the work. IR, XD and MG performed surgical procedure and analyzed and interpreted the patient data regarding the surgical management. JL and AM performed the ovarian cryopreservation and were major contributors in writing the manuscript. All authors read and approved the final manuscript.
